# A phone-based tobacco use cessation program for people living with HIV in Uganda and Zambia: study protocol for a randomized controlled trial

**DOI:** 10.1186/s13722-024-00438-w

**Published:** 2024-01-19

**Authors:** Heather Wipfli, Jim Arinaitwe, Fastone Goma, Lynn Atuyambe, David Guwatudde, Masauso Moses Phiri, Elizeus Rutebemberwa, Fred Wabwire-Mangen, Richard Zulu, Cosmas Zyambo, Kyra Guy, Ronald Kusolo, Musawa Mukupa, Ezekiel Musasizi, Joan S. Tucker

**Affiliations:** 1 Keck School of Medicine, Department of Preventative Medicine, University of Southern California, 2001 N. Soto Street, Los Angeles, CA 90033 USA; 2 School of Public Health, Makerere University, Centre for Tobacco Control in Africa, Kampala, Uganda; 3 School of Medicine, University of Zambia, Centre For Primary Care Research, Lusaka, Zambia; 4 School of Public Health, Department of Community Health and Behavioural Sciences, Makerere University, Kampala, Uganda; 5 Department of Epidemiology and Biostatistics, Makerere University School of Public Health, Kampala, Uganda; 6 School of Medicine, Department of Pathology and Microbiology, University of Zambia, Lusaka, Zambia; 7 School of Public Health, Department of Health Policy, Makerere University, Planning, and Management, Kampala, Uganda; 8 School of Public Health, Department of Community and Family Medicine, University of Zambia, Lusaka, Zambia; 9 RAND Corporation, Santa Monica, CA USA

**Keywords:** Tobacco, Addiction, Intervention, Text message, HIV, Sub sahran Africa

## Abstract

**Background:**

Nicotine replacement therapy (NRT) and short messaging service (SMS)-based tobacco cessation interventions have demonstrated effectiveness in reducing tobacco use in many populations, but evidence is needed on which tailored treatments are most efficacious in meeting the complex medical and psychosocial factors confronting people living with HIV (PLWH) in sub-Saharan Africa (SSA). This paper describes the protocol of a study to test the efficacy of both NRT and a tailored SMS-based tobacco use cessation intervention among PLWH in Uganda and Zambia.

**Methods:**

In a randomized controlled trial, 800 adult PLWH who use tobacco will be recruited by health care professionals at HIV treatment centers where they are receiving care. Participants will be randomized to one of the four study arms: (1) standard of care [SOC; brief clinician advice to quit combined with HIV education and information aimed at encouraging HIV treatment adherence (with no mention of tobacco) delivered via text messages]; (2) SOC + 12 weeks of NRT; (3) SOC + 6 weeks of SMS text messages to support quitting tobacco use (SMS); or (4) SOC + NRT + SMS. Participants will receive a cell phone and solar panel with power bank for charging the phone. The main outcome is cessation of tobacco use by study participants verified by urinary cotinine (< 15 ng/mL) at 6 months post-enrollment. As a secondary tobacco use outcome, we will measure 7-day point-prevalence abstinence (7 consecutive days of no tobacco use) measured by self-report and biochemically-verified at 4 weeks, 8 weeks, and 3 months post enrollment.

**Discussion:**

Our study will provide insight into the efficacy, feasibility and applicability of delivering tobacco cessation interventions through health care professionals combined with tailored tobacco cessation SMS text messaging in two countries with different tobacco use patterns, policy environments, and health care resources and provide needed information to providers and policymakers looking for cost-effective tobacco cessation interventions. The previously tested SMS-platform to be used in our study is uniquely positioned to be scaled in low- and middle-income countries worldwide, in which case evidence of even modest success in reducing the prevalence of tobacco consumption among PLWH could confer enormous health and economic benefits.

*Trial registration:* ClinicalTrials.gov Identifier NCT05487807. Registered August 4, 2022, https://clinicaltrials.gov/ct2/show/record/NCT05487807

## Background

AIDS-related morbidity and mortality among people living with HIV (PLWH) have decreased with the introduction of combination antiretroviral therapy (ART), but HIV remains among the world’s deadliest infectious diseases. In 2021, an estimated 650,000 people died from AIDS-related illnesses, with 51% of new HIV infections occurring in sub-Saharan Africa (SSA) [1]. Both Uganda and Zambia have significant HIV burdens, with Zambia having about double the prevalence of Uganda (10.8 vs. 5.2, respectively) [2]. Tobacco use among PLWH contributes substantially to the HIV burden, with an estimated 24% of AIDS-related deaths attributable to smoking [3]. Tobacco use among PLWH contributes to increased rate of progression to AIDS, poorer outcomes in HIV-associated opportunistic infections, increased risk of developing chronic diseases, and poorer adherence and response to ART [4–6]. Notably, PLWH in SSA have high rates of tuberculosis, which is exacerbated by smoking, making this region a particularly critical one for tobacco cessation efforts [7]. Similar to elsewhere, having HIV has been found to be associated with a greater likelihood of tobacco use in SSA with adjustment for demographic, socioeconomic, and sexual risk factors [8–10]. In Uganda, a study from 2014, which used salivary cotinine to identify tobacco users, found the prevalence of tobacco use among PLWH was twice that of the local background population (20% vs 10%) [11]. In Zambia, the 2018 Demographic and Health Survey found that the prevalence of tobacco use was higher among PLWH than non-PLWH for both women (4.4% vs. 2.4%) and men (27.8% vs. 18.7%) [12].

Research on tobacco cessation in PLWH indicates unique challenges, including higher rates of depressive symptoms and mental health issues, greater use of alcohol and illegal substances, lack of awareness about tobacco’s effects on HIV treatment, lower interest in quitting, and lower adherence to treatments [5, 13–17]. However, evidence suggests that HIV-positive smokers are receptive to quitting smoking and intention to quit smoking may increase upon the onset of HIV or an advancement in disease progression [18]. Tailored treatment approaches that meet the complex medical and psychosocial factors PLWH face, including education on how tobacco use impacts HIV prognosis, addressing depressive symptoms, and connecting patients to community support services, can improve outcomes among PLWH [19–22]. The medical infrastructure built around HIV treatment, including regular contact between HIV patients and health professionals, provides natural entry points for such sustained tobacco use cessation interventions. Prior rigorous research underscores the feasibility of integration of tobacco cessation services in resource-constrained HIV treatment centers [23–25].

There are multiple evidence-based cessation strategies that have been widely tested and found effective within the general population in high-income countries, including clinic-based tobacco cessation interventions such as physician-delivered tobacco cessation advice [26], group therapy [27], individual counseling [28], self-help materials [29], telephone counseling [30], and nicotine replacement therapy [31]. A handful of studies have been conducted in low- and middle-income countries (LMICs) [32] and in SSA focused on NRT and psychotherapy with mixed results [33]. A Cochrane Systematic Review also concluded that automated short text messaging (SMS) interventions were more effective than minimal smoking cessation support and text messaging added to other smoking cessation interventions was more effective than the other smoking cessation interventions alone [34]. A separate systematic review also concluded that smoking cessation support delivered by SMS increases quitting rates and was more effective at supporting cessation than smart phone smoking cessation treatment applications [35].

Recognizing the widespread use of mobile phones across socio-demographic groups in LMICs [36] and the potential of phones-based interventions to overcome communication and health care access barriers in LMICs [37, 38], WHO and the International Telecommunications Union launched the *Be He@lthy, Be Mobile* program in 2014 [39]. The program supports national scale-up and sustainability of mHealth programs related to non-communicable diseases, including a SMS-based program to help tobacco users quit [40]. To date, the tobacco cessation platform has been implemented in over 10 countries with positive results in regard to efficacy and acceptability [41]. For example, in India, the SMS-cessation platform enrolled over 1 million users within a year and achieved 19% successful quit rate at 4–6 months after completion of the program [42]. In 2017, WHO funded the Center for Tobacco Control in Africa to adapt and test the platform in Uganda among tuberculosis patients [43]. The resulting program, entitled Quit4Life, has been adapted, translated and is currently being tested in 7 districts in Uganda. While these study data are not yet published, numerous mHealth research studies conducted in Uganda and Zambia in other disease areas have found phone-based interventions are feasible, inexpensive, and viewed favorably by patients [44–46].

### The present study

While NRT and SMS-based tobacco cessation interventions have demonstrated effectiveness in reducing tobacco use in many populations, evidence is needed on which tailored treatments are most efficacious in meeting the complex medical and psychosocial factors confronting PLWH in SSA. We approach this gap with the first randomized controlled trial (RCT) to test the efficacy of both NRT and a tailored SMS-based tobacco use cessation intervention among PLWH in Uganda and Zambia. Our study will provide insight into the efficacy, feasibility and applicability of delivering tobacco cessation interventions through health care professionals at HIV treatment centers in two countries with different tobacco use patterns, policy environments, and health care resources and provide needed information to providers and policymakers looking for cost-effective tobacco cessation interventions. The previously tested SMS-platform to be used in our study is uniquely positioned to be scaled in LMICs worldwide, in which case evidence of even modest success in reducing the prevalence of tobacco consumption among PLWH could confer enormous health and economic benefits.

## Methods/Design

### Overview

**Fig. 1:**
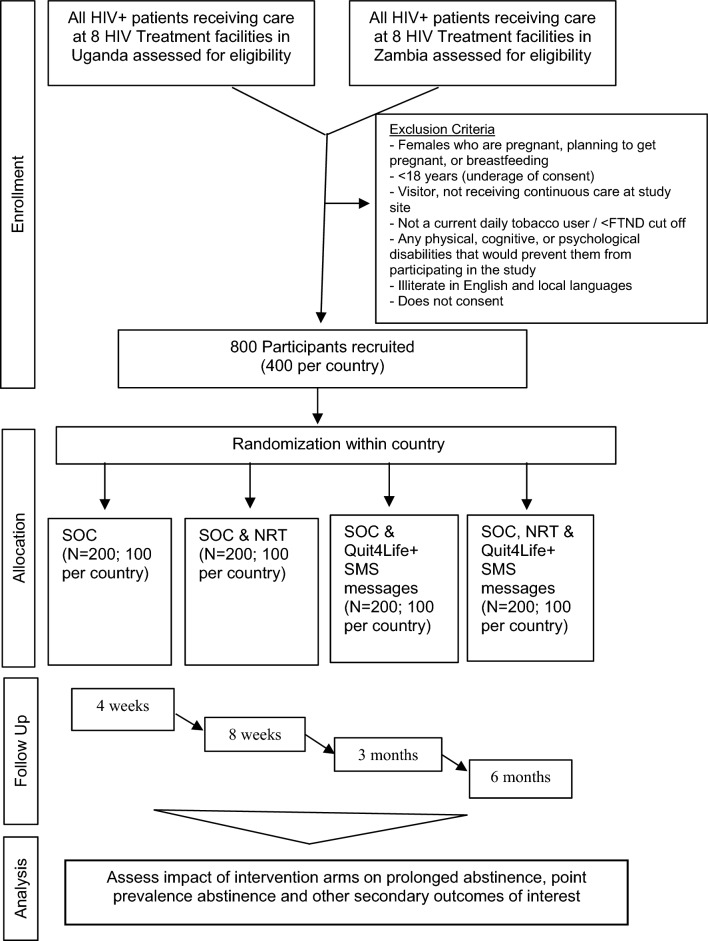
Randomized controlled study trial flow

Our primary goal is to carry out an RCT to test whether a text-based tobacco use cessation program adapted for PLWH who use tobacco in Uganda is more efficacious than brief clinician advice to quit – the current standard of care (SOC) and the provision of nicotine patches (Fig. 1). Specifically, 800 PLWH who use tobacco will be recruited from HIV treatment centers in two districts in Uganda and Zambia each and randomized to one of the following four study arms (described in more detail below): (1) Standard of Care (SOC); (2) SOC + NRT; (3) SOC + SMS; and (4) SOC + NRT + SMS. The main outcome of interest is cessation of tobacco use by study participants verified by urinary cotinine (< 15 ng/mL) at 6 months post-enrollment. This factorial design is efficient for assessing the effect of both nicotine patches and the adapted SMS program in a single trial with the possibility of saving both time and resources.

A core aim of this research is to generate data to inform the rapid scale-up of previously tested strategies, including mobile technology, for PWLH in LMICs. To achieve that, our study design tests interventions across and between diverse tobacco use patterns, policy environments, and health resources. While Uganda and Zambia are both SSA countries, they have differing HIV prevalence rates, tobacco use patterns, and tobacco control policy environments. For example, in 2015 Uganda passed comprehensive tobacco control legislation, while Zambia has struggled to push their legislation through parliament [47]. In site selection, we have considered regional prevalence of HIV, tobacco use patterns, and client load at HIV treatment centers. To meet the aims of the RCT, we chose two regions in each country with the highest tobacco use, allowing us to confidently recruit a sample size to more easily detect the desired effect size in case it exists.

### Participants and recruitment

Individuals will be eligible for the study if they are HIV + , use tobacco according to the definition below, are aged 18 years or older, and are receiving continuous HIV care from a treatment facility. Individuals will be ineligible for study participation if they: are currently pregnant or breastfeeding (or planning to do so in the next 6 months); have a physical or cognitive condition that would prevent them from study participation; or are illiterate in both English and the local language.

Participants will be recruited by health care professionals at HIV treatment centers where they are receiving care based on their HIV + status. Eligibility screening for the RCT will include completion of a questionnaire about current and past tobacco use, including intention to quit and/or past quit attempts. We will inquire about both combustible and non-combustible tobacco use, as there is a substantial amount of smokeless tobacco use (e.g., rolled, sniffed) in the districts that are the geographic focus of the study. We anticipate that e-cigarette use among our sample population will be rare. Tobacco use status will be biologically verified at baseline using urine cotinine dipsticks [48, 49]. The cotinine cut-off to detect current tobacco use will be > 15 ng/mL, as validated among African populations [50–52]. Although data are scarce, we suspect a substantial number of irregular smokers within the study catchment areas and level of dependence may also vary from what is typical in other countries [18, 53]. Consequently, typical measures used to identify participants for smoking cessation interventions, such as 10 cigarettes a day, may be inappropriate for this study. Therefore, in addition to self-reported and biologically-verified daily tobacco use, we will include a threshold level of nicotine dependence. We will train local recruiters to administer the Fagerstrom Test for Nicotine Dependence (FTND) [54] for both smoking and smokeless tobacco use [55]; individuals must have a score of 4 (the average score for a daily smoker [56]) or above to participate in the study.

Verbal consent will be obtained for completing the eligibility screener and eligible individuals will then be asked to provide written informed consent for RCT participation. Research staff will read the consent form aloud to the individual, who will follow along from their own copy (which will be available in both English and the local language), and answer any questions that the individual might have before signing the form. A Certificate of Confidentiality has been obtained for this project.

### Intervention setting

To facilitate the identification of PLWH that leverage existing HIV care infrastructure, we will carry out the study at HIV treatment centers in 16 sites located in two remote districts in Uganda and Zambia each [57–60]. In Uganda, the two districts selected are Arua located in the northern West-Nile region, and Moroto located in the northeast Karamoja region. West-Nile (Arua) is an agrarian society, whereas Karamoja region (Moroto) is mainly a nomadic society with strong social cultural ties. In Zambia, two districts in two provinces were selected: Mongu in the Western Province and Chipata in the Eastern Province. Mongu is in the western part and Chipata is in the eastern part of the country, both approximately 600 kms from Lusaka, the capital city. The predominant livelihood activity for the people in the Mongu study site is fishing in the flood planes of the Zambezi River, while on the other hand, people in the Chipata study sites are small scale farmers. To this extent, the two sites are different. However, Mongu (Western) and Chipata (Eastern) are similar in the sense that they are the highest two sites in Zambia in terms of HIV prevalence rates.

### SMS delivery

At enrollment, all participants will receive a project-provided mobile phone and a solar panel for charging the phone, as well as instruction on how to use the phone (including the use of security settings to alleviate privacy concerns). The phones will be basic text-based phones with no study identifiers. Such phones are very prevalent throughout the study areas and previous research with similar basic mobile phones has shown that they are effective for text message delivery and user friendly [61–63]. While mobile phone coverage is widespread in Uganda and Zambia, PLWH in our target communities represent among the most vulnerable individuals and thus least likely to have a personal phone; as such, due to the efficacy aims of this study providing phones will facilitate enrollment and expedite our results. 

The SMS text libraries will differ across intervention conditions. Participants in the SOC and SOC + NRT arms will receive 22 text messages focused specifically on HIV education and importance of adhering to HIV treatment regimen. These texts will not include an interactive component (e.g., no check-ins with participant; participant cannot request additional information). The HIV text messages were adapted from Uganda AIDS Commission (UAC) HIV messages library [64]. Participants in the SOC + SMS arm will receive the 22 SOC text messages, as well as up to 225 text messages focused on tobacco cessation: 164 standard text messages, 33 prompted questions (e.g., asking if they have quit), and 28 possible requested information texts. Table 1 provides the flow of when participants will receive the cessation messages throughout the program. These messages were either adapted from SmokefreeTXT or developed based on stakeholder feedback during the formative phase of this project, as described below. Finally, participants in the SOC + NRT + SMS arm receive the 22 SOC text messages, the up to 225 text messages focused on tobacco cessation, and up to 11 additional text focused on use of the nicotine patch (i.e., 5 standard text messages, 5 prompted questions, and 1 possible requested information text).

**Table 1:** Quit4Life + tobacco cessation-related text message flow (does not include SOC- or NRT-related texts)

Timepoint	Number of texts per day
Enrollment texts (Day -15)	12
Pre-quit texts (Days -14 to -1)	
14 to 8 days before quit day	2 texts
7 to 1 day before quit day	3 texts
Quit day texts (Day 0)	
Quit day	6 texts
Early post-quit texts (Days 1 to 14)	
1 day post-quit	3 texts
2–6 days post-quit	4 texts
7 day post-quit	5 texts
8–14 days post-quit	4 texts
Later post-quit texts (Days 15 to 42)	
15 to 28 days post-quit	2 to 3 texts
29 to 42 days post-quit	2 texts
Tobacco use check-ins	
Days 72, 132, and 222	1 text

### Description of intervention components

*Standard of Care (SOC)*. Participants in all four arms will receive the current minimum standard of care for tobacco cessation among PLWH in the study regions: brief clinician advice to quit delivered by staff at the HIV treatment center, combined with HIV education and information aimed at encouraging HIV treatment adherence (with no mention of tobacco) which will be delivered in this study via text messages.

#### Nicotine Replacement Therapy (NRT)

Participants in two of the four conditions (SOC + NRT; SOC + NRT + SMS) will receive up to a 12-week supply of nicotine patches, and clinician-delivered instructions on how to use the patch that follow package insert guidelines. The instructions will also include information on potential side effects of patch use. Dosing instructions will be tailored based on participants’ daily amount of tobacco use.

#### *Quit4Life* + *SMS Program (SMS)*

We followed World Health Organization recommendations for developing an mTobaccoCessation program for PLWH in Uganda and Zambia [39]. We first selected an existing SMS program that was publically available, evidence-based, and had been adapted for use in several countries outside the U.S.: SmokefreeTXT (http://www.smokefree.gov) [65]. In its original form, SmokefreeTXT is comprised of 125 standard text messages that are delivered over a 6-week period. These core messages focus on cessation strategies and support. In addition, there are periodic check-in texts to determine if participants are still using tobacco, as well as a pool of “on demand” texts that participants can receive to help them deal with cravings (text CRAVE), negative moods (text MOOD), or slips (text SLIP) during the quitting process. After selecting SmokefreeTXT for adaptation, we engaged in a formative phase of the project to obtain input from key stakeholders on what needed to be modified. Specifically, we conducted focus group discussions in Uganda and Zambia with health care providers (4 groups, 2 per country) and PLWH who used tobacco (8 groups, 4 per country) specifically for the purposes of adapting SmokefreeTXT for our target population [66]. Members of our research team from Uganda and Zambia, with combined expertise in tobacco cessation and HIV, also provided input on the adaptation process and resulting text library throughout the process.

SmokefreeTXT was adapted in four main respects: (a) modified existing texts to refer to tobacco use, rather than smoking specifically; (b) if possible, modified existing texts so that content was more appropriate and applicable to the local context in Uganda and Zambia; (c) deleted existing texts that could not be reasonably adapted to the local context; and d) added new texts to reflect content specifically tailored to PLWH in Uganda and Zambia that emerged from the formative research. Table 2 shows examples of additional domains that were added to the adapted SmokefreeTXT text library (e.g., basic education about health effects of tobacco use; information on HIV-related effects of tobacco use and quitting; socioemotional issues related to HIV-status that affect quitting; use of tobacco to decrease hunger; community norms that support tobacco use).

**Table 2:** Examples of domains added to SmokefreeTXT based on formative research

Domain	Example text(s)
Basic education about health effects of tobacco use	Whether smoked, chewed, rolled, or sniffed, tobacco contains nicotine. Tobacco is very addictive. When you quit you will break your addiction
Information on HIV-related effects of tobacco use and quitting	Using tobacco will not improve your CD4 count. In fact, tobacco use is associated with a poorer response to HIV treatment
Alcohol use is widespread and a trigger for tobacco use	Drinking alcohol makes it more difficult to quit tobacco. For the next few days avoid alcohol and see if that helps reduce tobacco use
Socioemotional issues related to HIV-status such as boredom/idleness, isolation, loneliness	Think of healthy ways to deal with stress & boredom instead of using tobacco. Engage in a hobby or visit a friend to keep yourself busy Loneliness can make quitting hard, but tobacco-free is the way to go. Reach out to a trusted friend to ask for their company & support
Tobacco is used to decrease hunger	Using tobacco may decrease your hunger, but it harms your body and can result in sickness and early death
Community norms support tobacco use (e.g., use is widespread and not considered harmful; not using is a sign of weakness or disrespect; using is linked to friendships; women often hide their tobacco use)	Some might say that not using tobacco shows weakness or disrespect. That’s not true. Quitting shows others that you can do the hard work to improve your health Your community may not be concerned about tobacco, but you now know how harmful it can be. Stay strong for you and your health Even when there is a lot of tobacco around you, it doesn’t mean that it is safe for you. Continue staying tobacco-free Tobacco use will not improve your social status. It affects your health & the health of those around you. Continue staying tobacco-free If you hide your tobacco use, it might help to tell a trusted friend why you are quitting and how they can support you
Religious guidance may help quitting	Some people get support for quitting by praying to God for guidance, support and strength to overcome tobacco. Try it if you think it will work for you

### Randomization and blinding

Prior to initiation of the trial, a reputable media company/agency not associated with implementation of the trial will be contracted to be responsible for: i) generating a randomization scheme/schedule using serial numbers from 1 through 400 for each country site using an appropriate STATA code; ii) randomly assigning enrolled participants to the study arms; and iii) sending the respective SMS messages per the assigned study arm to the participants. The media company/agency will keep the generated randomization schedule and manage the randomization of participants. When a participant is enrolled at any of the study enrollment sites, they will be assigned the next serial number which also corresponds to a telephone number. The media company/agency not associated with implementation of the trial will immediately be notified, who will then assign the study arm to the participant in a sequential order of enrollment per the randomization schedule. The agency will subsequently start sending the respective SMS messages to the participants per the study arm assigned. This way the participants, the clinic staff, and the study team members including the investigators will be blinded to the allocation of participants to any of the study arms. However, since the participants will interact with the clinic staff every time they come for treatment at the health facilities, it is possible that they may share with the staff the messages they have received; as such, the staff may be able to determine which study arm the participant was assigned. Therefore, blinding is only assured up to the time when participants begin receiving the study messages.

### Project monitoring and evaluation

Participants will receive prompts (close-ended questions that require a yes or no or numeric answers) on their phones periodically and at 6 months follow up to collect data about the study’s acceptability, feasibility, and impact. A subset of participants (*N* = 50) will be contacted by phone to provide more detailed feedback on their experience in the study. We will also collect feedback from participating clinic staff on the study through interviews (*n* = 30, 15 in each country) and focus groups (*n* = 4, 2 in each country). Key issues of concern with the clinic staff include ease of use and maintenance of the tablets that they will be asked to use for data collection, time it took to provide brief advice to quit and/or prescribe NRT and/or enroll a patient in the SMS program, acceptability of the program among patients, and whether providers felt the program improved their overall HIV treatment approach. We will track enrollment and retention trends and investigate any data inconsistencies that may reflect poor performance or deviations from the study procedures.

In addition, there will be an independent Data Safety and Monitoring Board (DSMB) composed of a clinician who is a specialist in HIV, a biostatistician, an expert in interventions studies and a community representative. The DSMB will monitor the safety of the participants, but also ensure that the study is conducted according to the protocol and will have access to the results on a regular basis so that they can take a decision at the earliest possible time when necessary. Specifically, the functions of the DSMB will be to review the research protocol, review the plans for data safety and monitoring, evaluate the progress of the intervention trial, review the data quality and timeliness as well as the accrual and retention. Other functions will include participants’ risk versus benefit and the performance of the trial sites.

In terms of adverse events, no adverse events or serious adverse events are expected from this study. However, it is possible that participants who have been heavy tobacco users may experience withdrawal symptoms. Participants will be informed that if they feel unwell, they should contact the study nurse immediately. Once contacted, the study nurse will fill the form for Adverse Event and refer the patient to the health care system. The participants will already be receiving treatment from the health care system but once they report some symptoms, this will be assessed to ensure that the participants are well taken care of. The particular people who will report adverse events will be told to come back for follow up even if they would have finished the treatment for another one month to ensure that the symptoms have disappeared. The study nurses will undergo training before the study in recording on the adverse events records form. Participants will be reporting to the health worker to pick their drug refills on treatment follow-up schedules but at the same time, the health worker will ask the participants whether they have any symptoms of discomfort. The health workers will be trained to support the patients with these symptoms but also refer them to more qualified personnel. All the health facilities that will be used are linked to the Regional Referral Hospitals where there are specialized doctors. These types of facilities have health workers who are trained to counsel patients and support them beyond the HIV clinics. The participants will be referred to these health workers for the necessary support.

### Reasons for and handling of withdrawals

In case a participant reports that s/he does not want to receive SMS messages again, the study team will ask the participant why they do not want to receive the SMS messages and then the study team will send the message to the SMS messaging platform to stop the messages being sent. The demographic characteristics of participants who choose to withdraw will be analysed and compared with those who have continued to the end to assess whether there is any difference. Among the questions asked from those who request to withdraw will be whether they are feeling any adverse symptoms to their health. In case they mention any adverse symptom, since they will have come to the health facility to collect their drugs or to report the problem, they will be put into the health system for clinical assessment and management. The clinic staff will fill the Adverse Event Form as determined by the National Drug Authority and this will be one of the forms to be reviewed by the DSMB alongside any subsequent outcome from the clinical assessment from the health facility where they are receiving treatment. The participants will be informed from the very beginning that when they feel any adverse event, they should immediately report to the study health personnel. 

### Termination of study

Termination of the study may occur if there are more adverse effects in one study arm compared to the others. This will be monitored by the DSMB regularly. The second possibility is when the sample size is reached before the 30 months scheduled for the data collection are finished. Once the target sample size is reached, study enrollment will be discontinued.

### Analytic Plan

#### Data management

Data will be collected at enrollment and at follow up appointments by trained clinic staff through a mobile-based application, REDCap (Research Electronic Data Capture), loaded onto a password protected tablet [67]. Data will be uploaded to the cloud daily through secure internet connections at the clinic sites to a REDCap webserver guarded by multiple firewall and intrusion detection systems. All electronic connections to the REDCap environment are encrypted. The data manager at the Center for Tobacco Control in Africa (CTCA) central coordination office will be responsible for downloading the data off the cloud into a secure MySQL database server behind the Makerere University firewall. REDCap implements authentication to validate the identity of end-users that log in to the system. The data manager at the CTCA central coordination office will be responsible for checking the accuracy and completeness of the data before entry into the database and following up with the relevant field team if any inconsistencies or problems are detected. Data will also be received from an independent media agency engaged by the study which contains a robust analytics framework to analyze the data collected, including how often participants receive and respond to prompts. The application interface allows for outside applications to interact with the data to carry out additional analyses. All data will be cleaned for final analysis by the CTCA data manager after it has been received.

#### Missing data

A number of measures will be implemented to minimize missing data. First, the survey instrument that will be pre-loaded on the data collection mobile tablets will be setup with data quality control measures including: i) data fields whose ranges can be pre-determined will be restricted so that entry of out of range values are not accepted; ii) data fields that must have an entry will also be restricted such that if left blank the person entering the data is alerted; and, iii) inconsistent data entries between different fields that can be pre-determined will not be accepted. Further, the Study Data Manager will on a daily basis conduct data checking by running frequencies of the data fields to try and identify any obvious anomalies, and if any is identified, he/she will alert the responsible study members so that they can correct it immediately or be prepared to correct it at the earliest interaction with the study participant.

#### Intent-to-treat Sample and Intervention Non-compliance

Analyses will use the standard intent-to-treat approach to examine the relative effects of the treatment arms on the outcomes of interest. Our intent-to-treat approach will analyze participants as belonging to the group they were randomized to, regardless of their compliance, because excluding those who do not use the SMS program would bias results in favor of the SMS conditions, increasing the probability of type I errors.

#### Statistical analyses

Multilevel logistic regression analysis will be used to assess differences in rates of tobacco use cessation between the study arms, and identification of the predictors of tobacco use cessation. For secondary outcomes that consider longitudinal data based on multiple follow ups and to adjust for the multi-level clustering per the study design, we will use either Generalized Estimating Equations or Logistic Mixed Effects Models to assess predictors. The level of significance will initially be set at p < 0.05, although given the number of outcomes of interest we will likely adjust for multiple comparisons using the Bonferroni correction [68]. Country and Region as indicators will be adjusted for as covariates, and also explored as an effect modifiers to allow for country-specific and region-specific effects, allowing us to explore how heterogeneity between and within countries and regions impacts the efficacy of the interventions and to what extent elements of the SMS-based intervention need to be tailored to address local, national and regional differences.

#### Measures

The primary tobacco use outcome for this efficacy trial is the proportion of study participants that have prolonged abstinence (i.e., no tobacco use from the target quit date through follow-up) at 3 and 6 months post enrollment measured by self-report and biochemically-verified as recommended by the Society for Research on Nicotine and Tobacco [69]. As a secondary tobacco use outcome we will measure 7-day point-prevalence abstinence (7 consecutive days of no tobacco use) measured by self-report and biochemically-verified at 4 weeks, 8 weeks, and 3 months post enrollment. The biomarker being assessed is urinary cotinine (< 15 ng/mL). We will also assess self-reported tobacco quit attempts and changes in tobacco use at 4 weeks, 8 weeks, and 3 months post enrollment. Finally, for those receiving nicotine patches, we will assess nicotine patch adherence at 4 weeks, 8 weeks, and 3 months post enrollment. Patch adherence will be assessed as the number of days the participant used a patch over the prior 4 week period, with adherence defined as using > 6 patches a week [70]. Self-reported patch use will be verified by a health worker who will conduct a patch count.

#### Power considerations

We assume that the main analysis will combine data from both countries (with proper adjustment for country). Given that the prevalence of tobacco use in the study area is estimated at 14.5%, we will assume that we will observe a minimal reduction in cessation in the SOC arm of only 2%, whereas in the intervention study arms we will observe a higher percentage of cessation in tobacco use. For example, if we observe a reduction in cessation down to 13.5% in the SOC arm, and a reduction in cessation down to 11.5% in the SOC + NRT arm, the effect size between these two arms would be 2%. Thus, we calculated the power of the study assuming a range of effect sizes; ∆ = 2%, 3%, 4%, 5%, 5.5%, and 6%. Below we summarize the estimated power to detect the difference in percentage of participants ceasing smoking between any two study arms being compared, assuming *n* = 200 per study arm (Uganda and Zambia combined). Thus, we will enroll 800 participants overall, 400 per country. Each intervention arm will enroll 200 participants (100 per country). Assuming a 5% loss to follow-up by month 6, the effective sample size per trial arm per country will be 95 participants.

As seen in Table 3, the trial will have more than 80% power to detect an effect size of at least 10.1% between any two study arms. Assuming a conservative 5% attrition within 6 months and hence *N* = 190 per trial arm, we expect to detect a 10.1% difference (i.e., from 14.2% to 4.1%) in the proportion of PLWH participants that quit smoking 6 months post intervention. All calculations are made for a 5% level of significance.

**Table 3:** Study power calculations

Effect size (∆): Difference in % of cessation between any 2 study arms (%)	Study power, given an effective sample size of 190 per study arm (%)
4.0	28.1
6.0	18.1
8.0	60.0
10.0	79.3
10.1	80.1

### Dissemination

Our dissemination strategy will target tobacco and HIV stakeholders including Ministry of Health and other government officials, health care organizations, health facility managers, HIV health care providers, tobacco control professionals, non-governmental organizations engaged in HIV and tobacco control, and PLWH. Our investigators will publish study results through peer-reviewed journals and scientific conferences; generate fact sheets and policy briefs; and disseminate our results through websites, social media, television and radio. We will share the study results in the participating Districts and will host a workshop in the final year with regional stakeholders to share the study results and promote tobacco use cessation programming for PLWH throughout SSA. WHO will assist in disseminating the study findings to other LMICs. The study will disseminate the pre-and post-evaluation results, detailing quit rates, periodic review results and Quit4Life + approach effectiveness. These pieces of information will be disseminated through websites, newsletters, factsheets, social media platforms, journals and conferences. Data from the study will be shared using an aggregated format. Shared data will not have any identifiers. Apart from dissemination, data will be accessed only on request.

## Discussion

### Potential limitations and anticipated challenges

It is a limitation of the study that participants may share the information and texts they receive between the intervention arms and this source of contamination may not be easy to control. However, many of the texts are specific to the participant’s stage of quitting (e.g., pre-quit preparation vs. quit day vs. maintenance), and these may not be relevant to the other person s/he is sharing with. Another potential limitation is that recharging the phone batteries may be a challenge and participants may not get access to the messages in an optimum manner. We will attempt to minimize this problem by providing participants with solar panels with power banks for charging their phones. Other limitations include shared use of the phone and low literacy rates in Moroto. In both cases incoming messages may be missed and or shared through a third party introducing potential for misunderstanding or bias.

In terms of anticipated challenges, these include high turnover of clinic staff, low morale and interest in the study’s execution among the local team, and poor compliance among study participants. Health workers may not be interested in participating in additional tobacco-related advising as this may be considered unnecessary and viewed as additional workload to the already overburdened staff. To counter this, we will seek highly motivated health care providers, emphasize the role tobacco use cessation plays in improving HIV treatment outcomes, and provide a monthly stipend to compensate for the additional workload and keep the staff motivated. We may face recruitment challenges as there may be hesitancy to self-report tobacco use, challenges identifying participants with sufficiently high FTND scores, and stigma arising from their HIV status and tobacco use may result in lower willingness to participate/poor retention. To counter this, our interviewers will be trained in techniques on probing and direct observations of the respondent’s reactions. Other challenges include political disruptions and natural disasters (e.g. COVID-19) which will require flexibility and resilience on behalf of the study team. Procurement of resources (patches, cotinine tests, phones, tablets and solar chargers) can be challenging, but the study team has experience navigating these bureaucracies. Finally, partner communication can be a challenge; however, the study team has worked together well for a number of years and already have weekly virtual meetings to ensure constant communication.

### Conclusion

Quit4Life + is a culturally-adapted SMS-based program to help PLWH in Uganda and Zambia quit tobacco use. Given the widespread use of mobile phones across socio-demographic groups in LMICs, and its potential to overcome communication and health care access barriers, it is critical to evaluate the acceptability, feasibility, and effectiveness of this approach for reducing tobacco use among PLWH in LMICs. If found to be effective, this SMS-platform is uniquely positioned to be scaled in LMICs worldwide.

## Data Availability

Once collected, deidentified data from this study will be available from the corresponding author on reasonable request one year after all aims of the project are completed. Requestors of data will be asked to complete a data-sharing agreement that provides for (1) a commitment to using the data only for research purposes and not to identify any individual participant; (2) a commitment to securing the data using appropriate computer technology; and (3) a commitment to destroying or returning the data after analyses are completed.

## Abbreviations

AIDS: Acquired immunideficiency syndrome
ART: Anti-retroviral therapy
CTCA: Center for Tobacco Control Africa
HIV: Human immunodeficiency virus
NRT: Nicotine replacement therapy
PLWH: People living with HIV
SMS: Short message/messaging service
SOC: Standard of care
SSA: Sub-Saharan Africa
